# *Pneumocystis jirovecii* Diversity in Réunion, an Overseas French Island in Indian Ocean

**DOI:** 10.3389/fmicb.2020.00127

**Published:** 2020-02-07

**Authors:** Solène Le Gal, Gautier Hoarau, Antoine Bertolotti, Steven Negri, Nathan Le Nan, Jean-Philippe Bouchara, Nicolas Papon, Denis Blanchet, Magalie Demar, Gilles Nevez

**Affiliations:** ^1^Groupe d’Étude des Interactions Hôte-Pathogène (GEIHP) EA 3142, Université d’Angers-Université de Brest, Angers, France; ^2^Laboratory of Mycology and Parasitology, CHRU de Brest, Brest, France; ^3^Department of Microbiology, CHU La Réunion, Saint Pierre, France; ^4^Department of Infectious Diseases, CHU La Réunion, Saint Pierre, France; ^5^Laboratory of Mycology and Parasitology, Andrée Rosemon Hospital, Cayenne, French Guiana; ^6^Equipe EA3593 - Ecosystèmes Amazoniens et Pathologie Tropicale, Université de Guyane, Cayenne, French Guiana

**Keywords:** *Pneumocystis jirovecii*, genotypes, Réunion, *Pneumocystis* pneumonia, multilocus sequence typing (MLST), French Guiana, France

## Abstract

Data on *Pneumocystis jirovecii* characteristics from the overseas French territories are still scarce whereas numerous data on *P. jirovecii* genotypes are available for metropolitan France. The main objective of the present study was to identify *P. jirovecii* multilocus genotypes in patients living in Réunion and to compare them with those identified using the same method in metropolitan France and in French Guiana. Archival *P. jirovecii* specimens from immunosuppressed patients, 16 living in Réunion (a French island of the Indian ocean), six living in French Guiana (a South-American French territory), and 24 living in Brest (Brittany, metropolitan France) were examined at the large subunit rRNA (mtLSUrRNA) genes, cytochrome *b* (*CYB*), and superoxide dismutase (*SOD*) genes using PCR assays and direct sequencing. A total of 23 multi-locus genotypes (MLG) were identified combining mtLSUrRNA, *CYB*, and *SOD* alleles, i.e., six in Reunionese patients, three in Guianese patients, and 15 in Brest patients. Only one MLG (mtLSU1-*CYB*1-*SOD*2) was shared by Reunionese and Guianese patients (one patient from each region) whereas none of the 22 remaining MLG were shared by the 3 patient groups. A total of eight MLG were newly identified, three in Réunion and five in Brest. These results that were obtained through a retrospective investigation of a relatively low number of *P. jirovecii* specimens, provides original and first data on genetic diversity of *P. jirovecii* in Réunion island. The results suggest that *P. jirovecii* organisms from Réunion present specific characteristics compared to other *P. jirovecii* organisms from metropolitan France and French Guiana.

## Introduction

*Pneumocystis jirovecii* is an opportunistic and transmissible fungus responsible for severe pneumonia *Pneumocystis* pneumonia (PCP) in immunocompromised patients. PCP remains the most frequent AIDS-defining illness in human immunodeficiency virus (HIV)-infected patients in metropolitan France (Cazein et al., 2015) and the West French Indies (Martinique and Guadeloupe) whereas in French Guiana, another French region of Americas, PCP occupies the fifth position of AIDS causes. PCP is also the most frequent AIDS-defining illness in Réunion, a French island of the Indian Ocean, located close to Capricorn tropic, 600 km from east coast of Madagascar (Cire Océan Indien, 2015). HIV-infection incidence is higher in the French regions of Americas than in metropolitan France whereas its incidence is lower in Réunion (Cazein et al., 2015). PCP is also a severe disease in other immunosuppressed patients who are not infected with HIV, such as patients treated with immunosuppressive and/or cytostatic therapies (Roux et al., 2014).

**FIGURE 1:**
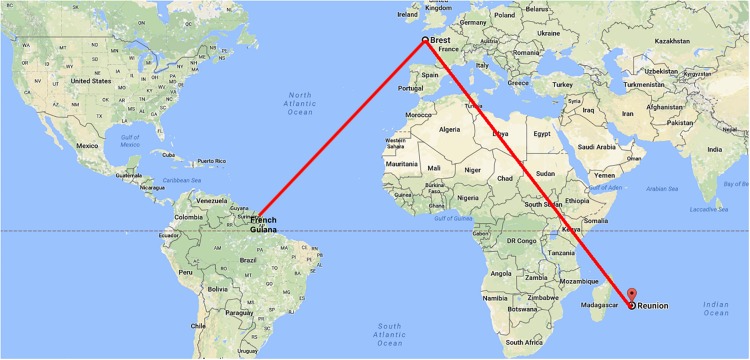
Geographical locations of Reunion Island, French Guiana and Brest.

Data on *P. jirovecii* characteristics from the overseas French territories are still scarce. Indeed, there is only one report on this topic, which concerned *P. jirovecii* genotypes in French Guiana (Le Gal et al., 2015) located 7,000 km from metropolitan France whereas there are no data on genomic characteristics of *P. jirovecii* from Réunion, located 9,300 km from metropolitan France and 12,000 km from French Guiana (Figure 1). Conversely, numerous data on *P. jirovecii* genotypes are available for metropolitan France (Nevez et al., 2003; Totet et al., 2003; Le Gal et al., 2013; Maitte et al., 2013; Gits-Muselli et al., 2015; Alanio et al., 2016; Desoubeaux et al., 2016; Charpentier et al., 2017; Vindrios et al., 2017). In this context, the main objective of the present study was to identify *P. jirovecii* multilocus genotypes in patients living in Réunion and to compare these genotypes with those identified using the same method in patients living in metropolitan France or French Guiana.

## Materials and Methods

### *Pneumocystis jirovecii* Specimens and Patients

Seventeen *P. jirovecii* specimens from 16 patients [sex ratio M/F 13/3, median age 52 years (limits, 30–72 years)] who developed PCP and who were monitored at South Réunion Island University Hospital, were retrospectively studied. The 16 patients were diagnosed with PCP from March 2015 through June 2017. Ten patients had hematological malignancies, five patients were HIV-infected, and one patient had non-X histiocytosis.

Six *P. jirovecii* specimens from six patients [sex ratio M/F 1/5; median age 33 years (range, 30–57)] monitored at Andrée Rosemon Hospital, Cayenne, French Guiana, were also studied retrospectively. The six patients were HIV-infected and developed PCP from November 2011 through October 2012.

Twenty-four *P. jirovecii* specimens from 24 patients [sex ratio M/F 16/8, median age 64 years (limits, 33–84 years)] monitored at Brest University Hospital, Brest, Brittany, metropolitan France, were also analyzed retrospectively. These 24 patients were diagnosed with PCP from January 2013 through June 2017. Clinical and biological data of the three patient groups are summarized in Table 1 and detailed in Supplementary Table S1. Data on Guianese patients were previously published elsewhere (Le Gal et al., 2015). Data on Brest patients were previously published in part elsewhere (Nevez et al., 2020).

**TABLE 1:** Characteristics of the three groups of patients from whom *Pneumocystis jirovecii* specimens were genotyped.

	**La Réunion**	**French Guiana**	**Brest (metropolitan France)**
No. of patients	16	6	24
Sex ratio (M/F)	13/3	1/5	16/8
Median age (range)	52 (30–72)	33 (30–57)	64 (33–84)
Period of PCP diagnosis (mo/yr)	03/2015–06/2017	11/2011–10/2012	01/2013–06/2017
Presentation of *Pneumocystis* infection (No. of patients)	PCP (16)	PCP (6)	PCP (24)
Techniques of *Pneumocystis* detection in pulmonary specimens (No. of patients)	Musto stain (7), PCR (16)^a^	Wright-Giemsa (5), IFA (4)^b^	IFA (15), PCR (24)^c^
Risk factors for *Pneumocystis* infection (No. of patients)	Hematological malignancy (10), HIV infection (5), non-X histiocytosis (1)	HIV infection (6)	Cancer (7), HIV infection (6), hematological malignancy (5), renal transplantation (2), immunosuppressive treatment (2), lymphopenia (2)

*F, female; HIV, Human Immunodeficiency Virus; IFA, immunofluorescence assay (MonofluoKit *Pneumocystis*, Bio-Rad, Marnes-La-Coquette, France); M, male; PCP, *Pneumocystis* pneumonia; PCR, polymerase chain reaction. ^*a*^The PCR assay was performed as described by Hoarau et al. (2017). All specimens were positive for *P. jirovecii* detection using PCR whereas only 7 out 16 were positive using Musto stain. ^*b*^5 out of 6 specimens were positive for *P. jirovecii* detection using Wright – Giemsa stain whereas 4 out of 6 were positive using IFA. ^*c*^The PCR assay was performed as described by Le Gal et al. (2017). All specimens were positive for *P. jirovecii* detection using PCR whereas only 15 were positive using IFA.*

Patients of the three groups had undergone a bronchoalveolar lavage (BAL) or induced sputum procedure to investigate pulmonary symptoms and/or fever. *P. jirovecii* had initially been detected in specimens by microscopy using Musto stain, Wright Giemsa stain, and/or an indirect immunofluorescence assay (MonofluoKit *Pneumocystis*, Bio-Rad, Marnes-La-Coquette, France), and/or PCR assays amplifying the mtLSUrRNA gene as described elsewhere (Hoarau et al., 2017; Le Gal et al., 2017). Extracted DNAs from the three patient groups were stored at −80°C until typing.

The study was non-interventional, and therefore did not require inform consents and ethical approval according to French laws and regulations (CSP Art L1121e1.1).

### *Pneumocystis jirovecii* Typing

Extracted DNAs of BAL and induced sputum samples were examined for *Pneumocystis* genotyping based on unilocus and multilocus sequence typing (MLST) methods. Three loci, mtLSUrRNA, cytochrome *b* (*CYB*) and superoxide dismutase (*SOD*) genes were analyzed, as we previously described, using direct sequencing (Vindrios et al., 2017). Consensus sequences were aligned with reference sequences [GenBank accession numbers M58605 (mtLSUrRNA), AF074871 (*CYB*) and KT592355 (*SOD*)] (Sinclair et al., 1991; Walker et al., 1998; Singh et al., 2017) using the BioEdit software with the Clustal^®^ W program. MtLSUrRNA alleles were named using the nomenclature described previously by Beard et al. (2000), *CYB* and *SOD* alleles were named using the nomenclature described previously by Esteves et al. (2010) and Maitte et al. (2013). According to Struelens (1996), the discriminatory power which was determined using Hunter index (H) (Hunter, 1990) was considered good if higher than 0.95. To avoid contamination, each step of the PCR assays was performed in different areas of the laboratory with different sets of micropipettes. Mix reagents were prepared in a laminar flow cabinet. To monitor for possible contamination, negative controls were included in each experiment and PCR round.

The Maximum Likelihood method implemented in MEGA (version 7.0.26) was used to reconstruct a phylogenetic tree based on the Hasegawa-Kishino-Yano model (Kumar et al., 2016). Substitution model was determined by Bayesian Information Criterion in jModelTest 0.1.1 (Posada, 2008). Bootstrap values for internal branches were generated from 1,000 replicates. MtLSUrRNA (209 bp), *CYB* (563 bp) and *SOD* (380 bp) sequences were concatenated and aligned to a reference sequence (SeqRef mtLSU CYB SOD). This reference sequence corresponds to a 209-bp portion of mtLSUrRNA reference sequence (M58605) concatenated with a 563-bp portion of *CYB* reference sequence (AF074871) and a 380-bp portion of *SOD* reference sequence (KT592355) (Sinclair et al., 1991; Walker et al., 1998; Singh et al., 2017).

Relatedness between *Pneumocystis* MLGs was evaluated with the minimum spanning tree (MST) method using GrapeTree, a free web browser application implementing Kruskal’s algorithm and Edmonds’ algorithm (Zhou et al., 2018). MLGs were treated as multistate categories based on an infinite allele model, i.e., all changes are equally likely.

### Nucleotide Sequence Accession Numbers

The nucleotide sequences of the new *CYB* allele sequences with changes at scoring positions have been deposited in GenBank under accession numbers MN602710 and MN602711.

## Results

*Pneumocystis* genotyping results are detailed in Table 2. MtLSUrRNA sequences were obtained for the 16 Reunionese patients (17 samples), the six Guianese patients, and the 24 Brest patients. Five alleles were identified, considering the mtLSU alleles previously described elsewhere (Beard et al., 2000; Esteves et al., 2010; Table 2). MtLSU1 was the most frequent allele in Reunionese patients (7 patients; 43.7%). MtLSU3 was the most frequent allele in Guianese patients (3 patients; 50%). MtLSU1, mtLSU2 and mtLSU3 were the most frequent alleles in Brest patients, these three genotypes being equally detected (seven patients each; 29.2%). MtLSU1, mtLSU2 and mtLSU3 were shared by the three patients’ groups while mtLSU4 was shared by Reunionese and Brest patients. MtLSU5 was identified only in one Reunionese patients (6.2%). The presence of more than one allele was observed in seven Reunionese patients (43.7%), two Guianese patients (33.3%) and six patients from Brest (25%). The Hunter index for mtLSU genotyping was evaluated to 0.76.

**TABLE 2:** Genotypes of *Pneumocystis jirovecii* identified in patients developing *Pneumocystis* pneumonia from Réunion, French Guiana and Brest.

**Patient code**	**Underlying conditions**	**mtLSU allele**	***CYB* allele**	***SOD* allele**	**Multilocus genotype**
R1	ALL	mtLSU1 + mtLSU2	*CYB*8 + *CYB*10	Mixed	Mixed
R2	HIV infection	mtLSU1 + mtLSU4	*CYB*3 + *CYB*1	Mixed	Mixed
R3	AML	mtLSU2 + mtLSU3	*CYB*5	*SOD*1	Mixed
R4	AML	mtLSU1	ND	ND	ND
R5	Myeloma	mtLSU4	*CYB*11	*SOD*2	mtLSU4-*CYB*11-*SOD*2
R6	Non-X histiocytosis	mtLSU4	*CYB*6	*SOD*1	mtLSU4-*CYB*6-*SOD*1
R7	ALL	mtLSU4	*CYB*11	*SOD*2	mtLSU4-*CYB*11-*SOD*2
R8	HIV infection	mtLSU1 + mtLSU2 + mtLSU3	*CYB*1	Mixed	Mixed
R9	Polycythemia vera	mtLSU1 + mtLSU2	*CYB*1 + *CYB*8	*SOD*1	Mixed
R10	HIV infection	mtLSU4	*CYB*1	ND	ND
		mtLSU1 + mtLSU4	*CYB*1	Mixed	Mixed
R11	Myeloma	mtLSU3	*CYB*10	*SOD*1	mtLSU3-*CYB*10-*SOD*1
R12	Lymphoma	mtLSU2	*CYB*1	ND	ND
R13	AML	mtLSU5	*CYB*3	ND	ND
R14	HIV infection	Mixed	Mixed	*SOD*1 + *SOD*4	ND
R15	ALL	mtLSU3	*CYB*1	*SOD*4	mtLSU3-*CYB*1-*SOD*4
R16	HIV infection	mtLSU1	*CYB*1 + *CYB*6	*SOD*2	mtLSU1-*CYB*1-*SOD*2 + mtLSU1-*CYB*6-*SOD*2
G1	HIV infection	mtLSU2	*CYB*1	*SOD*2	mtLSU2-*CYB*1-*SOD*2
G2	HIV infection	Mixed	Mixed	Mixed	Mixed
G3	HIV infection	mtLSU1 + mtLSU3	*CYB*1	*SOD*2	mtLSU1-*CYB*1-*SOD*2 + mtLSU3-*CYB*1-*SOD*2
G4	HIV infection	mtLSU3	*CYB*1	*SOD*2	mtLSU3-*CYB*1-*SOD*2
G5	HIV infection	mtLSU2	*CYB*1	*SOD*2	mtLSU2-*CYB*1-*SOD*2
G6	HIV infection	mtLSU3	*CYB*1	*SOD*2	mtLSU3-*CYB*1-*SOD*2
B1	HIV infection	mtLSU2	*CYB*2	*SOD*1	mtLSU2-*CYB*2-*SOD*1
B2	Cancer	mtLSU4	*CYB*1	*SOD*1	mtLSU4-*CYB*1-*SOD*1
B3	Cancer	mtLSU1	*CYB*5 + *CYB*8	*SOD*2	mtLSU1-*CYB*5-*SOD*2 + mtLSU1-*CYB*8-*SOD*2
B4	Immunosuppressive treatment	mtLSU1	*CYB*2	*SOD*2	mtLSU1-*CYB*2-*SOD*2
B5	HIV infection	mtLSU1 + mtLSU4	*CYB*2	*SOD*1	mtLSU1-*CYB*2-*SOD*1 + mtLSU4-*CYB*2-*SOD*1
B6	AML	mtLSU1	*CYB*8	*SOD*2	mtLSU1-*CYB*8-*SOD*2
B7	Lymphopenia	mtLSU2	Mixed	Mixed	Mixed
B8	Lymphopenia	mtLSU2	*CYB*8	*SOD*1	mtLSU2-*CYB*8-*SOD*1
B9	Myeloma	mtLSU2	*CYB*7 + *CYB*1	Mixed	Mixed
B10	Lymphoma	mtLSU3	*CYB*1	*SOD*1	mtLSU3-*CYB*1-*SOD*1
B11	Myeloma	mtLSU4	*CYB*1	*SOD*1	mtLSU4-*CYB*1-*SOD*1
B12	Renal Transplant Recipient	mtLSU4	*CYB*2	*SOD*1	mtLSU4-*CYB*2-*SOD*1
B13	Cancer	Mixed	*CYB*1 + *CYB*2	*SOD*2	Mixed
B14	Cancer	mtLSU1	*CYB*1	Mixed	Mixed
B15	HIV infection	mtLSU3	*CYB*6	*SOD*1	mtLSU3-*CYB*6-*SOD*1
B16	Immunosuppressive treatment	Mixed	*CYB*1 + *CYB*6	*SOD*2	Mixed
B17	HIV infection	mtLSU1 + mtLSU3	*CYB*1	*SOD*1 + *SOD*4	Mixed
B18	Cancer	mtLSU2	*CYB*1	Mixed	Mixed
B19	Lymphoma	mtLSU2	*CYB*2	Mixed	Mixed
B20	Cancer	mtLSU1 + mtLSU3	*CYB*1	*SOD*1	mtLSU1-*CYB*1-*SOD*1 + mtLSU3-*CYB*1-*SOD*1
B21	HIV infection	mtLSU3	*CYB*5	*SOD*1	mtLSU3-*CYB*5-*SOD*1
B22	Cancer	mtLSU3	ND	*SOD*2	ND
B23	Renal Transplant Recipient	mtLSU4	*CYB*2	*SOD*1	mtLSU4-*CYB*2-*SOD*1
B24	HIV infection	mtLSU2 + mtLSU3	*CYB*2	*SOD*2	mtLSU2-*CYB*2-*SOD*2 + mtLSU3-*CYB*2-*SOD*2

*F, female; HIV, Human Immunodeficiency Virus; M, male; ALL, acute lymphoblastic leukemia; AML, acute myeloblastic leukemia; mtLSUrRNA, mitochondrial large subunit ribosomal RNA; *CYB*, cytochrome *b*; *SOD*, superoxide dismutase; mixed, allele mix due to the presence of several different alleles that could not be identified; ND, not determined. Patients and samples are identified with a letter (R for Reunionese patients, G for Guianese patients and B for Brest patients) followed by a number.*

*CYB* sequences were obtained from 15 out of 16 Reunionese patients (16 samples), as well as from the 6 Guianese patients and 23 out of 24 patients from Brest. Considering the *CYB* alleles previously described elsewhere (Esteves et al., 2010; Maitte et al., 2013), seven already known alleles (*CYB*1, *CYB*2, *CYB*3, and *CYB*5 to *CYB*8) and two new alleles (*CYB*10 and *CYB*11) were identified (Table 2), *CYB*1 being the most frequent in the three patient groups [seven Reunionese patients (46.7%), five Guianese patients (83.3%), 10 Brest patients (43.5%)]. The new *CYB*10 allele differs from *CYB*2 by a change from C to T residue at scoring position 279, and the new *CYB*11 allele differs from *CYB*1 by a change from C to T residue at scoring position 742. *CYB*1 was shared by the three populations. In contrast, *CYB*3, *CYB*10, and *CYB*11 were identified only in Reunionese patients [three (20%), three (20%) and two (13.3%) patients, respectively] while *CYB*2 and *CYB*7 were detected only in Brest patients [eight (37.8%) patients and one (4.3%) patient, respectively]. The presence of more than one allele was observed in five Reunionese patients (33.3%), one Guianese patient (16.7%) and five patients from Brest (21.7%). The Hunter index for *CYB* genotyping was evaluated to 0.76.

*SOD* sequences were obtained from 13 out of the 16 Reunionese patients, as well as from the six Guianese patients and the 24 patients from Brest. Considering the *SOD* alleles previously described elsewhere (Esteves et al., 2010; Maitte et al., 2013), three alleles were identified (Table 2), *SOD*1 being the most frequent in Reunionese and Brest patients (five (38.5%) and 12 (50%) patients, respectively) and *SOD*2 the most frequent in Guianese patients [five patients (83.3%)]. *SOD*2 was identified in the three patient groups. The presence of more than one allele was observed in five Reunionese patients (38.5%), one Guianese patient (16.7%) and six patients from Brest (25%). The Hunter index for *SOD* genotyping was evaluated to 0.59.

Combining mtLSUrRNA, *CYB*, and *SOD* alleles, 23 multi-locus genotypes (MLG) were identified (Table 2). MtLSU4-*CYB*11-*SOD*2, mtLSU3-*CYB*1-*SOD*2, and mtLSU4-*CYB*2-*SOD*1 were the most frequent MLG in Reunionese patients (two patients, 16.7%), Guianese patients (three patients, 50%), and Brest patients (three patients, 13%), respectively. Only one MLG (mtLSU1-*CYB*1-*SOD*2) was shared by the Reunionese and the Guianese patients (one patient from each region, 8.3 and 16.7% respectively) whereas the 22 remaining MLG were not shared by the three patient groups. The presence of more than one genotype was observed in seven Reunionese patients (58.3%), two Guianese patients (33.3%) and 12 patients from Brest (52.2%). The Hunter index for MLST was evaluated to 0.978.

**FIGURE 2:**
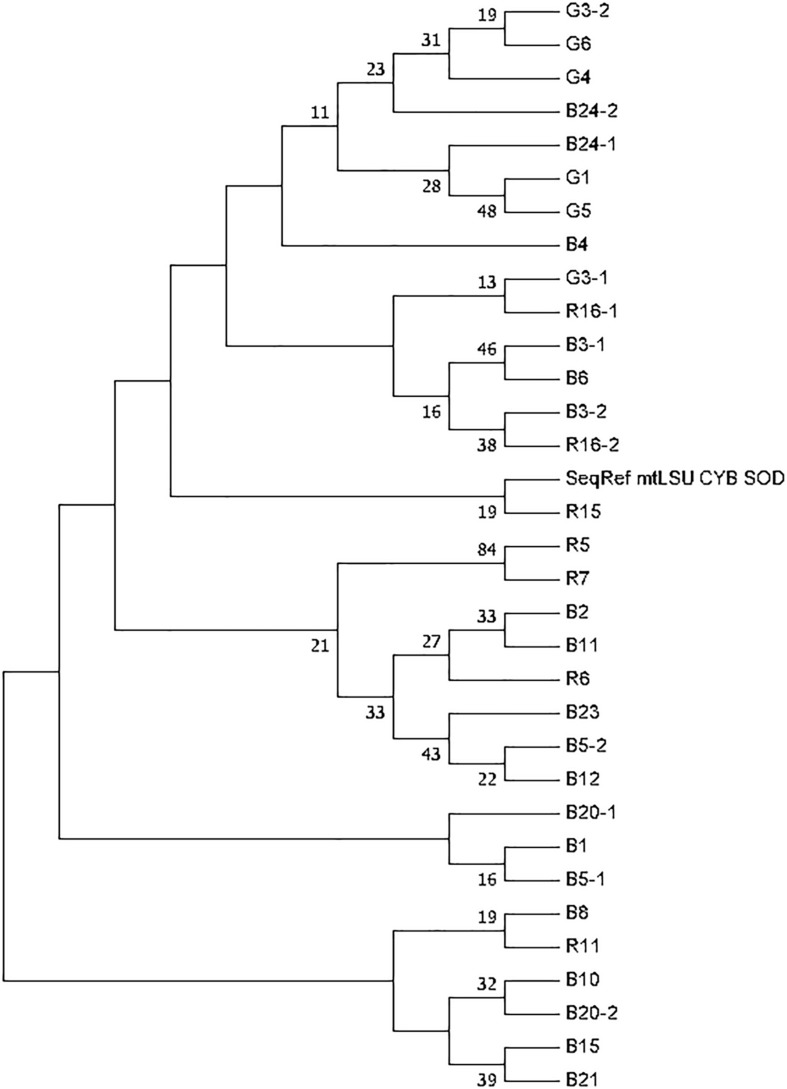
Phylogenetic tree generated based on *Pneumocystis jirovecii* MLST genotypes. The tree was constructed on the basis of concatenated loci (mtLSUrRNA, *CYB*, and *SOD*). Phylogenetic analysis used the Maximum Likelihood method based on the Hasegawa-Kishino-Yano model. The bootstrap consensus tree was inferred from 1,000 replicates. Branches corresponding to partitions reproduced in less than 50% bootstrap replicates are collapsed. The percentage of replicate trees in which the associated taxa clustered together in the bootstrap test are shown next to the branches. Only bootstrap values >10% are shown. Initial tree(s) for the heuristic search were obtained automatically by applying Neighbor-Join and BioNJ algorithms to a matrix of pairwise distances estimated using the Maximum Composite Likelihood (MCL) approach, and then selecting the topology with superior log likelihood value. The analysis involved 33 nucleotide sequences. There were a total of 1152 positions in the final dataset. Evolutionary analyses were conducted in MEGA7. Patients and samples are identified with a letter (R for Reunionese patients, G for Guianese patients and B for Brest patients) followed by a number.

The phylogenetic tree was generated based on sequence analysis of mtLSUrRNA, *CYB*, and *SOD* alleles through the MLST approach (Figure 2). The analysis showed that some genotypes detected in patients from metropolitan France and the Réunion island were close despite the rarity of MLST genotype sharing. Nonetheless, the bootstrap values were low (<50).

**FIGURE 3:**
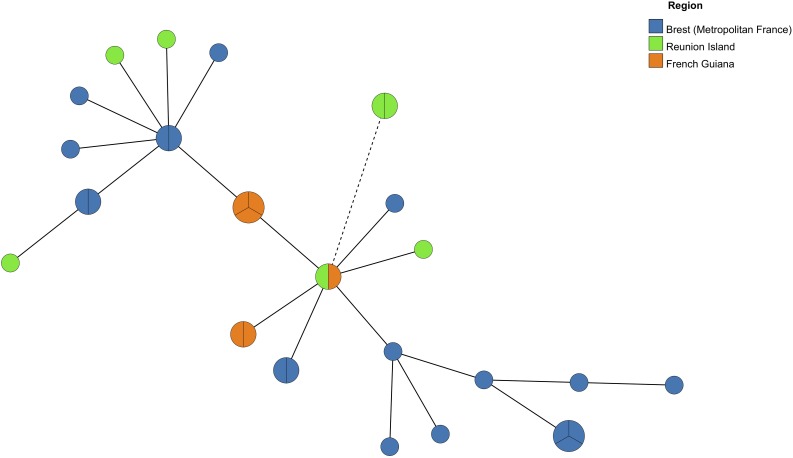
Minimum Spanning Tree analysis of *Pneumocystis* isolates from six Reunionese patients, five Guianese patients and 15 Brest (Metropolitan France) patients harboring one multilocus genotype (MLG) (one allele per locus) or two MLGs (two alleles for one locus). Each circle corresponds to one MLG. The size of the circles is correlated with the number of isolates possessing the corresponding MLG. The color of the circles indicates the origin region of the *Pneumocystis* isolates. A continuous connecting line corresponds to one different allele separating two MLGs. A dashed connecting line corresponds to two different alleles separating two MLGs.

As well, MST analysis revealed that most of *Pneumocystis* isolates from the three geographic regions were close and belonged to the same genetic cluster since they had a single allelic mismatch with at least one other isolate (Figure 3). However, two isolates from Réunion who shared the same MLG (mtLSU4-*CYB*11-*SOD*2) were more distant from the other isolates since they had two allelic mismatches with the closest isolate.

## Discussion

In this study, the first data on *P. jirovecii* genotypes in patients from Réunion, a French region in the Indian Ocean, were obtained. Using an unilocus approach, we identified mtLSUrRNA, *CYB*, and *SOD* common alleles in the three patient populations (Réunion, French Guiana, and Brest), suggesting that *P. jirovecii* organisms from Réunion, French Guiana and Metropolitan France share common characteristics. Nonetheless, some alleles were identified only from one patient population, e.g., mtLSU5, *CYB*10, and *CYB*11 were identified only from Reunionese patients.

We chose to analyze mtLSUrRNA, *CYB*, and *SOD* loci because this MLST scheme is highly discriminant as described by Maitte et al. (2013). Indeed, in the present study, the discriminatory power of our technique, based on the Hunter index, was evaluated to 0.978, which is considered to be good, i.e., >0.95, one criterion of those described by the ESCMID to validate a typing method (Struelens, 1996). The sequencing was performed using Sanger method, which is easy to be performed. However, it could be less sensitive to detect and identify alleles in a mixture than PCR-SSCP or high-throughput methods, such as ultra-deep sequencing or multiplex PCR-Single-Base-Extension (Hauser et al., 2001; Esteves et al., 2011, 2016; Alanio et al., 2016; Charpentier et al., 2017). In the present study mixed alleles were identified in 15 out of 46 patients (32.6%) for mtLSUrRNA, in 11 out of 44 patients (25%) for CYB, and in 12 out of 43 patients (27.9%) for SOD. Considering MLG analysis, mixed MLGs were identified in 21 out of 42 patients (50%). These rates are lower than those observed in studies using PCR-SSCP (Hauser et al., 2001) or molecular high-throughput methods, such as ultra-deep sequencing (Alanio et al., 2016; Charpentier et al., 2017), in which rates of mixed genotypes may reach 85% for nuclear loci and even 92% for mitochondrial loci. However, this potential weakness of our method should not question our analysis based on major genotypes. Moreover, the method we used is suitable for examining samples with low fungal loads (Vindrios et al., 2017; Nevez et al., 2018, 2020).

The alleles *CYB*10 and *CYB*11 identified in Reunionese patients were newly described in this study. Allele *CYB*10 differs from allele *CYB*2 described by Esteves et al. (2010) by the substitution from C to T residue at position 279, which is a silent mutation. However, this allele corresponds to haplotype 13 as described by Charpentier et al. (2017) using another nomenclature. It was identified in two patients from Grenoble, metropolitan France. Allele *CYB*11 differs from allele *CYB*1 by having a T residue at position 742. This substitution from C to T represents a non-synonymous mutation leading to the substitution from leucyl to phenylalanyl residue at position 275 (L275F). This mutation located at the quinol oxidation (Qo) site of the mitochondrial cytochrome bc1 may induce potential atovaquone resistance of *P. jirovecii.* Indeed, atovaquone which is used as second line treatment of PCP or PCP prophylaxis, is an analog of ubiquinone that binds the Qo site. This mutation, but not this allele, has previously been described by Kazanjian et al. (2001) among *P. jirovecii* isolates from patients with past history of atovaquone exposure. It is noteworthy that the two Reunionese patients, who were infected with cytochrome *b* mutant *P. jirovecii* organisms, were effectively subjected to atovaquone prophylaxis in a context of hematological malignancy (myeloma for one patient, and T-cell acute lymphoblastic leukemia for the other), at the time of PCP diagnosis. This observation was consistent with that we recently reported concerning the selection of cytochrome *b* mutants in heart transplant recipients in a context of PCP case clusters and the use of atovaquone prophylaxis (Argy et al., 2018).

In this study, allele mtLSU5 was identified only from one Reunionese patient. This infrequent allele has been previously identified in metropolitan patients from Brest and Lille (de Armas et al., 2012; Le Gal et al., 2015) and in a Guianese patient (patient G2 in the present study) (Le Gal et al., 2015) using cloning instead of direct sequencing of the mtLSUrRNA sequences. Thus, this allele does not represent a specific characteristic of *P. jirovecii* organisms from Réunion island.

There are available data on genotyping of *P. jirovecii* isolates from adult patients or children who lived in Zimbabwe or Mozambique (Africa), two countries relatively close to Réunion and from other adults or children who lived in Cuba (West Indies), a country relatively close to French Guiana (Miller et al., 2003, 2005; de Armas et al., 2012; Monroy-Vaca et al., 2014; Esteves et al., 2016). This geographical proximity deserves *P. jirovecii* genotype comparison. Considering the results of unilocus typing, mtLSU3 allele is the most frequent allele in adults from Cuba as well as in adults from French Guiana (de Armas et al., 2012), whereas mtLSU2 is the most frequent allele in children from Cuba (Monroy-Vaca et al., 2014). To the best of our knowledge, no information on *SOD* and *CYB* alleles in adults from Cuba is available. Conversely, in children, *CYB*1 and *SOD*1 are the most frequent alleles in Cuba whereas alleles *CYB*1 and *SOD*2 are the most frequent alleles in adults in French Guiana. No data on *P. jirovecii* genotyping in children from French Guiana are available.

MtLSU3 and mtLSU1 are the most frequent alleles in adults from Zimbabwe and Reunion respectively (Miller et al., 2003). In the same patient population, *SOD*2 and *SOD*1 are the most frequent alleles in Zimbabwe and Reunion respectively (Miller et al., 2003). Conversely, in children from Mozambique the most frequent allele is mtLSU2 and/or mtLSU5 (considering that information on scoring nucleotide position 248 is lacking) (Esteves et al., 2016). No data on *CYB* alleles from Zimbabwe and Mozambique, whichever patient population, are available.

Finally, due to differences in characteristics of the studied patient populations (adults vs. children, numbers of patients) and analyzed loci, it remains difficult to draw a conclusive analysis of this genotype comparison.

A total of 23 MLG were identified. It is noteworthy that 22 were not shared by the three patient group whereas only one MLG was shared by two of the three patient groups. Thus, the results of MLG analysis, due to the high discriminatory power of the method (Hunter index, 0.978), allow to discriminate *P. jirovecii* isolates into three different groups according to the geographic origin of the patients. Nonetheless, among the five MLG identified only in Reunionese patients, two (mtLSU1-*CYB*6-*SOD*2 and mtLSU3-*CYB*1-*SOD*4) has already been reported in patients from metropolitan France and Portugal (Esteves et al., 2010; Maitte et al., 2013). Conversely, the three other MLG (mtLSU3-*CYB*10-*SOD*1, mtLSU4-*CYB*11-*SOD*2, and mtLSU4-*CYB*6-*SOD*1) were reported for the first time in the present study. It is noteworthy that the MLG mtLSU4-*CYB*11-*SOD*2 consists in the combination of mtLSU4 allele, *SOD*2 allele and the newly described allele *CYB*11 which might have been selected in the course of atovaquone prophylaxis. Be that as it may, taken together, these results suggest that *P. jirovecii* organisms from Réunion island may present specific characteristics.

Three MLG were identified in Guianese patients; all of them have already been reported in patients from metropolitan France or Portugal (Esteves et al., 2010; Maitte et al., 2013; Desoubeaux et al., 2016; Charpentier et al., 2017). These results are not consistent with those previously obtained using another genotyping method (Le Gal et al., 2015), which suggested that specific genotypes and consequent specific characteristics of *P. jirovecii* organisms may exist in French Guiana. This could be explained by the fact that more data are available in literature on MLG combining mtLSUrRNA, *CYB*, and *SOD* alleles than on MLG combining ITS, *DHPS*, and mtLSUrRNA alleles, the method we used previously.

Fifteen MLG were identified in Brest patients, of which ten have already been reported in patients from metropolitan France and Portugal (Esteves et al., 2010; Maitte et al., 2013; Desoubeaux et al., 2016; Charpentier et al., 2017; Vindrios et al., 2017) whereas five were reported for the first time. These five MLG (mtLSU1-*CYB*2-*SOD*2, mtLSU1-*CYB*3-*SOD*1, mtLSU1-*CYB*5-*SOD*2, mtLSU2-*CYB*2-*SOD*2, and mtLSU3-*CYB*2-*SOD*2) may represent specific characteristics of *P. jirovecii* organisms in Brittany, western France. Likewise, the three MLG identified in Réunion island may represent specific characteristics of *P. jirovecii* organisms in this French overseas island. However, the results of the MST suggest that *P. jirovecii* organisms from the three French regions are closely related and belong to the same genetic cluster, excepting two isolates from Réunion. These two isolates share the same new MLG (mtLSU4-*CYB*11-*SOD*2) (see above).

Finally, a total of eight MLG were newly identified, three in Réunion and five in Brest, suggesting that specific characteristics in these two French regions, located 9,300 km apart may exist. However, genotyping results should not be too conclusive considering the low number of patients for whom MLG were identified in this study (six Reunionese patients, five Guianese patients, 15 patients from Brest). Moreover, the dates of *P. jirovecii* sampling in the three geographical regions were not identical, which may represent a bias of enrolment. Likewise, the underlying diseases of the patients were not strictly similar since Guianese patients were all HIV-infected contrary to Reunionese patients and patients from Brest, these disparities representing a bias as well. Furthermore, available data on MLG combining mtLSUrRNA, *CYB*, and *SOD* alleles are still limited since only four studies from France and one from Portugal were based on these sequences (Esteves et al., 2010; Maitte et al., 2013; Desoubeaux et al., 2016; Charpentier et al., 2017; Vindrios et al., 2017). Moreover, the results of phylogenetic tree and MST analysis are poorly informative. Be that as it may, despite these possible limitations, the present study brings original and first data on genetic diversity of *P. jirovecii* organisms from Réunion island and its comparison with other very distant French regions.

## Data Availability Statement

The datasets generated for this study can be found in the GenBank database (accession numbers: MN602710 and MN602711).

## Ethics Statement

Ethical review and approval was not required for the study on human participants in accordance with the local legislation and institutional requirements. Written informed consent for participation was not required for this study in accordance with the national legislation and the institutional requirements.

## Author Contributions

SL and GN analyzed the DNA sequences and wrote the manuscript. GH and AB performed *P. jirovecii* detection, provided *P. jirovecii* specimens from Réunion, and analyzed patients’ characteristics. SN and NL performed in part the genotyping. J-PB and NP contributed to the discussion and correction of the manuscript. DB and MD performed *P. jirovecii* detection and provided *P. jirovecii* specimens, and analyzed patients’ characteristics from French Guiana.

## Conflict of Interest

The authors declare that the research was conducted in the absence of any commercial or financial relationships that could be construed as a potential conflict of interest.
