# Supplementary material for: Pneumocystis jirovecii Diversity in Réunion, an Overseas French Island in Indian Ocean
Source: Front Microbiol. 2020 Feb 7;11:127. doi: 10.3389/fmicb.2020.00127 (PMC7019000; doi:10.3389/fmicb.2020.00127)
Supplement: Supplementary file 1 [file Table_1.docx]

Table S1. Characteristics of Reunionese, Guianese and French metropolitan patients who developed *Pneumocystis* pneumonia and for whom *Pneumocystis jirovecii* specimens were genotyped.

| Patient  code | Sex | Age | Underlying conditions | Blood CD4+ T cell count (10^6^/L) | Date of PCP diagnosis  (mo/yr) | Type of pulmonary specimen | Techniques of *P. jirovecii* detection in pulmonary specimens |
| --- | --- | --- | --- | --- | --- | --- | --- |
| R1 | M | 72 | ALL | - | 03/2015 | BAL | Musto stain; PCR |
| R2 | M | 34 | HIV | 34 | 06/2015 | BAL | Musto stain; PCR |
| R3 | M | 59 | AML | - | 07/2015 | BAL | PCR |
| R4 | F | 30 | AML | - | 12/2015 | BAL | PCR |
| R5 | M | 50 | Myeloma | - | 12/2015 | BAL | Musto stain; PCR |
| R6 | M | 58 | Non-X histiocytosis | - | 01/2016 | BAL | PCR |
| R7 | M | 48 | ALL | - | 01/2016 | BAL | PCR |
| R8 | M | 58 | HIV | 13 | 02/2016 | BAL | Musto stain; PCR |
| R9 | F | 50 | Polycythemia vera | - | 02/2016 | BAL | PCR |
| R10 | M | 62 | HIV | 4 | 06/2016 | BAL | Musto stain; PCR |
|  |  |  |  |  | 07/2016 | BAL | Musto stain; PCR |
| R11 | M | 42 | Myeloma | - | 09/2016 | BAL | PCR |
| R12 | M | 69 | Lymphoma | - | 10/2016 | BAL | PCR |
| R13 | M | 63 | AML | - | 02/2017 | BAL | PCR |
| R14 | M | 50 | HIV | 9 | 06/2017 | BAL | Musto stain; PCR |
| R15 | F | 54 | ALL | - | 06/2017 | BAL | PCR |
| R16 | M | 40 | HIV | 52 | 06/2017 | BAL | Musto stain; PCR |
| G1 | F | 33 | HIV | 5 | 11/2011 | BAL | Wright-Giemsa; IFA |
| G2 | F | 30 | HIV | 3 | 01/2012 | BAL | Wright-Giemsa |
| G3 | F | 33 | HIV | 25 | 02/2012 | BAL | Wright-Giemsa |
| G4 | M | 57 | HIV | 61 | 05/2012 | BAL | IFA |
| G5 | F | 33 | HIV | 9 | 10/2012 | BAL | Wright-Giemsa; IFA |
| G6 | F | 47 | HIV | 9 | 09/2012 | BAL | Wright-Giemsa; IFA |
| B1 | M | 34 | HIV | 10 | 01/2013 | BAL | IFA; PCR |
| B2 | M | 73 | Cancer | - | 03/2013 | BAL | IFA; PCR |
| B3 | M | 81 | Cancer | - | 09/2013 | BAL | IFA; PCR |
| B4 | M | 39 | Immunosuppressive treatment | 319 | 03/2014 | BAL | IFA; PCR |
| B5 | M | 42 | HIV | 131 | 08/2014 | BAL | IFA; PCR |
| B6 | F | 49 | AML | - | 09/2014 | BAL | IFA; PCR |
| B7 | F | 84 | Lymphopenia | - | 01/2015 | BAL | IFA; PCR |
| B8 | M | 80 | Lymphopenia | - | 02/2015 | BAL | IFA; PCR |
| B9 | M | 70 | Myeloma | - | 02/2015 | BAL | IFA; PCR |
| B10 | F | 70 | Lymphoma | - | 01/2016 | Sputum | PCR |
| B11 | F | 57 | Myeloma | 1648 | 02/2016 | BAL | PCR |
| B12 | M | 74 | Renal Transplant Recipient | 52 | 02/2016 | BAL | IFA; PCR |
| B13 | M | 60 | Cancer | - | 02/2016 | BAL | PCR |
| B14 | F | 69 | Cancer | - | 04/2016 | Sputum | PCR |
| B15 | F | 55 | HIV | 19 | 06/2016 | BAL | IFA; PCR |
| B16 | F | 69 | Immunosuppressive treatment | - | 08/2016 | BAL | PCR |
| B17 | M | 53 | HIV | 68 | 08/2016 | Sputum | PCR |
| B18 | F | 54 | Cancer | - | 09/2016 | BAL | PCR |
| B19 | M | 58 | Lymphoma | - | 09/2016 | BAL | IFA; PCR |
| B20 | M | 76 | Cancer | - | 09/2016 | BAL | PCR |
| B21 | M | 65 | HIV | 35 | 10/2016 | BAL | IFA; PCR |
| B22 | M | 66 | Cancer | - | 11/2016 | BAL | PCR |
| B23 | M | 64 | Renal Transplant Recipient | - | 03/2017 | BAL | IFA; PCR |
| B24 | M | 33 | HIV | 187 | 06/2017 | BAL | IFA; PCR |

Patients and samples are identified with a letter (R for Reunionese patients, G for Guianese patients and B for Brest patients) followed by a number. M, male; ALL, acute lymphoblastic leukemia; PCP, Pneumocystis pneumonia; BAL, bronchoalveolar lavage; PCR, polymerase chain reaction; HIV, Human Immunodeficiency Virus; AML, acute myeloblastic leukemia; F, female; IFA, immunofluorescence assay (MonofluoKit Pneumocystis, Bio-Rad, Marnes-La-Coquette, France).
